# Increased risk of peptic ulcer in patients with early-onset cataracts: A nationwide population-based study

**DOI:** 10.1371/journal.pone.0207193

**Published:** 2018-11-09

**Authors:** Ning-Yi Hsia, Yi-Yu Tsai, Cheng-Li Lin, Chun-Chi Chiang

**Affiliations:** 1 Department of Ophthalmology, China Medical University Hospital, Taichung, Taiwan; 2 School of Medicine, China Medical University College of Medicine, Taichung, Taiwan; 3 Management Office for Health Data, China Medical University Hospital, Taichung, Taiwan; 4 Department of Public Health, China Medical University College of Public Health, Taichung, Taiwan; National Yang-Ming University Hospital, TAIWAN

## Abstract

Early-onset cataracts (EOC) are associated with an increased inflammatory response; therefore, a potential risk of other inflammatory diseases, like peptic ulcer, may be related. This study investigated the risk of peptic ulcer after being diagnosed with EOC. Retrospective claims data from the Taiwan National Health Insurance Research Database were analyzed. Study subjects comprised patients with EOC (International Classification of Diseases, 9th Revision, Clinical Modification [ICD-9- CM] codes 366.00, 366.01, 366.02, 366.03, 366.04, 366.09, 366.17 and 366.18), aged 20–55 years and newly diagnosed between 2000 and 2010 (n = 1910), and a comparison cohort without the disease (n = 7515). Both cohorts were followed up until 2010 to estimate the incidences of peptic ulcer. We used the Poisson regression model to compare incidence rate ratios and the 95% confidence interval (CI). Cox proportional hazards regression was used to assess the hazard ratio (HR) of peptic ulcer associated with EOC. The overall incidence rate of peptic ulcer was higher in the EOC cohort than in the comparison cohort (10.3 vs 7.68 per 1000 person-years) with an adjusted HR of 1.33 (95% CI = 1.05, 1.69). The present study suggests that patients with EOC are at an increased risk of being diagnosed with peptic ulcer in subsequent years.

## Introduction

Cataract is reduced transparency in the lens of the eye and is the leading cause of vision loss in the world [1–5]. Cataracts usually affect people older than 60 years; however, early-onset cataract (EOC) can also develop in subjects younger than 55 years [6]. In normal aging, the oxidation of lens nuclear components increases, and this reduces its transparency. In addition to oxidative stress in the lens, the incidence of cataract is higher in the population that is more exposed to sunlight, cigarette smoking, and inflammatory status which were related to increased intraocular inflammation. [7, 8]. Cataract formation is also associated with decreased anti-inflammatory function and a reduction in the concentration and activities of anti-oxidants in the lens [9–16]. Hence, insufficient anti-oxidative and anti-inflammatory function might be the causes of the early formation of cataracts.

Peptic ulcer is a common disease, presenting as a defect in the upper gastrointestinal mucosa that extends into deeper layers of the gut wall. There are known definitive risk factors for peptic ulcer and they are preventable [17]. *Helicobacter pylori* is a major risk factor for peptic ulcer and incites an inflammatory response. In the majority of infected people remain asymptomatic but susceptible people may progress to peptic ulcer. Host factor also had an important role and it thought to be related to the patient’s ability to express the inflammatory mediators and the function of anti-oxidation and anti-inflammation [18–20]. In consideration of the preventability of peptic ulcer and subsequent sequelae, it seems reasonable to identify vulnerable groups at risk of developing peptic ulcer, particularly among younger people. Therefore, using a nationwide database, this study sought to determine whether there was an association between EOC and the risk of peptic ulcer.

## Materials and methods

### Data source

The Taiwan government launched the National Health Insurance (NHI) program in 1995, and it now covers more than 99% of Taiwan's population. The Longitudinal Health Insurance Database 2000 (LHID2000) is one of the databases in the larger National Health Insurance Research Database (NHIRD). The LHID2000 is composed of 1 million subjects randomly selected from the NHI program. The database provides information including the sex, birth date, disease codes, and medical records of the 1 million subjects. The identification numbers of the patients have been re-encoded to protect patient privacy. This study was approved by the Institutional Review Board, China Medical University and the Hospital Research Ethics Committee (IRB permit number: CMUH-104-REC2-115).

### Sampled participants

Patients who were newly diagnosed with EOC (ICD-9-CM codes 366.00, 366.01, 366.02, 366.03, 366.04, 366.09, 366.17, and 366.18) from January 1, 2000 to December 31, 2010 were the case study group, and the date of diagnosis was the index date. We excluded patients who had been diagnosed with peptic ulcer disease (ICD-9-CM code 531–533), received UGI endoscope (NHI order code: 28016C) and proton-pump inhibitors (ATC code: A02BC01, A02BC02, A02BC03, A02BC04, A02BC05) prior to the index date. The comparison group was composed of patients who had no record of being diagnosed with EOC in the LHD2000. The index date for comparison patients was randomly appointed a month and day with the same index year of the matched EOC cases. The exclusion criterion for the comparison group was the same as that for the case group. The comparison group was matched with the case group by age (every 5-year span), gender, the number of visits to clinics within 1 year of the index date, and index year.

### Outcome, relevant variables and comorbidities

The outcome of the study was peptic ulcer disease (ICD-9 code 531–533), the coding of procedure of upper gastrointestinal endoscopy (NHI order code: 28016C), and the use of proton-pump inhibiters (ATC code: A02BC01, A02BC02, A02BC03, A02BC04, A02BC05), and the end of the study was the date the patient was diagnosed with peptic ulcer disease or the end of 2011. We considered hypertension (ICD-9-CM codes 401−405), diabetes mellitus (ICD-9-CM code 250), chronic kidney disease (ICD-9-CM codes 580–589), coronary artery disease (ICD-9-CM codes 410–414), alcohol-related illness (ICD-9-CM 291, 303, 305, 571.0, 571.1, 571.2, 571.3, 790.3, A215, and V11.3), chronic obstructive pulmonary disease (COPD) (ICD-9-CM 491, 492, and 496), anxiety (ICD-9-CM code 300.00), depression (ICD-9-CM codes 296.2–296.3, 300.4, 311), gastroesophageal reflux disease (ICD-9-CM codes 530.11, 530.81), and cirrhosis (ICD-9-CM 571.2, 571.5, and 571.6) as comorbidities in this study. Treatment variables included systemic non-steroidal anti-inflammatory drug (NSAID) (ATC code: M01A), and systemic steroid use (ATC code: H02A).

### Statistical analysis

The Chi-squared test was used to analyze differences in categorical variables, and Student’s *t*-test was used for continuous variables. The incidence rate of peptic ulcer disease was calculated by person-years. The hazard ratio (HR) and 95% confidence interval (CI) for the risk of peptic ulcer disease were estimated by univariate and multivariate Cox proportional hazard regression models. The variables of age and sex, and comorbidities of hypertension, diabetes mellitus, chronic kidney disease, coronary artery disease, alcohol-related illness, chronic obstructive pulmonary disease, anxiety, depression, gastroesophageal reflux disease and cirrhosis were the adjusted variables in the multivariate Cox model. The Kaplan-Meier curve showed the cumulative incidence of peptic ulcer disease for both groups, and the differences between the two groups were tested using the log rank test. We further discussed the joint effects of EOC and risk factors for peptic ulcer disease on being diagnosed as having peptic ulcer disease. In this case, the adjusted variables of the multivariate Cox model were age and sex. The data analysis for this study was performed using SAS statistical software (Version 9.4 for Windows; SAS Institute, Inc., Cary, NC, USA). A p-value less than 0.05 was considered statistically significant.

## Results

There were 1910 patients in the case group and 7515 patients in the comparison group for this study (Table 1). Using the Chi-squared test, we found that the distributions of gender and age stratification of the two groups did not differ, but the distribution of age was different. The mean age of the case group was 0.4 year older than that of the comparison group. The case group had a higher proportion of comorbidities of hypertension, diabetes mellitus, chronic kidney disease, coronary artery disease, alcohol-related illness, and cirrhosis, and medication of steroid than the comparison group.

**Table 1:** Demographic characteristics and comorbidities of patients with and without early-onset cataract.

Variables	Early-onset cataract		*p*-value
	No	Yes	
	N = 7515	N = 1910	
Sex	N (%)	N (%)	0.94
Female	3847(51.2)	976(51.1)	
Male	3668(48.8)	934(48.9)	
Age, mean (SD)	45.7(7.49)	46.1(7.51)	0.04^#^
Stratified by age			0.93
20–34	4623(61.5)	1173(61.4)	
35–55	2892(38.5)	737(38.6)	
The number of visits to clinics within 1 year of the index date, mean (SD)	21.9(17.2)	23.7(23.5)	0.001
Comorbidities			
Hypertension	1584(21.1)	531(27.8)	<0.001
Diabetes mellitus	470(6.25)	357(18.7)	<0.001
Chronic kidney disease	58(0.77)	54(2.83)	<0.001
Coronary artery disease	591(7.86)	198(10.4)	<0.001
Alcohol-related illness	316(4.20)	103(5.39)	0.02
Chronic obstructive pulmonary disease	418(5.56)	121(6.34)	0.19
Anxiety	612(8.14)	148(7.75)	0.57
Depression	415(5.52)	99(5.18)	0.56
Gastroesophageal reflux disease	144(1.92)	39(2.04)	0.72
Cirrhosis	57(0.76)	33(1.73)	<0.001
Medication			
NSAID	4865(64.7)	12224(64.1)	0.59
Steroid	893(11.9)	304(15.9)	<0.001

Chi-Square Test

^#^: Two-sample T-test

Table 2 shows the HRs of peptic ulcer disease between the case group and the comparison group; the patients were stratified by sex, age, comorbidities, and medications in this table. Patients with any of the comorbidities in Table 1 were placed in the comorbidity group. Patients with EOC had a higher risk of peptic ulcer disease (adjusted HR = 1.33, 95% CI = 1.05–1.69). We found that patients with EOC had an increased risk of developing peptic ulcer disease in most of the stratifications, including younger than 34 years (adjusted HR = 1.37, 95% CI = 1.00–1.88), and with comorbidity (adjusted HR = 1.77, 95% CI = 1.28–2.45).

**Table 2:** Comparison of incidence and hazard ratio of peptic ulcer disease stratified by sex, age and comorbidity between patients with and without early-onset cataract.

	Early-onset cataract							
	No			Yes				
Variables	Event	PY	Rate^#^	Event	PY	Rate^#^	Crude HR (95% CI)	Adjusted HR^†^ (95% CI)
All	279	36311	7.68	93	8999	10.3	1.34(1.06, 1.70) *	1.33(1.05, 1.69)*
Sex								
Female	134	18785	7.13	43	4680	9.19	1.28(0.91, 1.81)	1.30(0.92, 1.85)
Male	145	17526	8.27	50	4319	11.6	1.40(1.02, 1.94) *	1.37(0.99, 1.91)
Stratified by age								
≤34	157	22604	6.95	54	5598	9.65	1.39(1.02, 1.89)*	1.37(1.00, 1.88)*
35–55	122	13706	8.90	39	3402	11.5	1.29(0.90, 1.84)	1.28(0.88, 1.86)
Comorbidity‡								
No	180	23675	7.60	36	4922	7.31	0.96(0.67, 1.37)	0.97(0.68, 1.39)
Yes	99	12636	7.83	57	4078	14.0	1.78(1.29, 2.47)***	1.77(1.28, 2.45)***
Medication								
NSAID								
No	115	15646	7.35	35	3791	9.23	1.25(0.86, 1.83)	1.20(0.81, 1.78)
Yes	164	20665	7.94	58	5209	11.1	1.40(1.04, 1.89)*	1.46(1.07, 1.98)*
Steroid								
No	260	32863	7.91	76	7798	9.75	1.23(0.95, 1.59)	1.23(0.95, 1.60)
Yes	19	3448	5.51	17	1201	14.2	2.56(1.33, 4.92)**	2.51(1.27, 4.96)**

Rate#: incidence rate, per 1,000 person-years; Crude HR: crude hazard ratio

Adjusted HR†: multivariate analysis including age, sex, the number of visits to clinics within 1 year of the index date, and comorbidities of hypertension, diabetes mellitus, chronic kidney disease, coronary artery disease, alcohol-related illness, chronic obstructive pulmonary disease, anxiety, depression, gastroesophageal reflux disease and cirrhosis, and medication of NSAID and steroid.

Comorbidity‡: Patients with any one of the comorbidities of hypertension, diabetes mellitus, chronic kidney disease, coronary artery disease, alcohol-related illness, chronic obstructive pulmonary disease, anxiety, depression, gastroesophageal reflux disease and cirrhosis were classified as the comorbidity group

*p<0.05

**p<0.01

***p<0.001

The EOC cohort with NSAID use was at a 1.46-fold increased risk of peptic ulcer compared with the non-EOC cohort with NSAID use (adjusted HR = 1.46, 95% CI = 1.07–1.98), and the EOC cohort with steroid use was at a 2.51-fold increased peptic ulcer risk compared with the non-EOC cohort with steroid use (adjusted HR = 2.51, 95% CI = 1.27–4.96).

**Fig 1:**
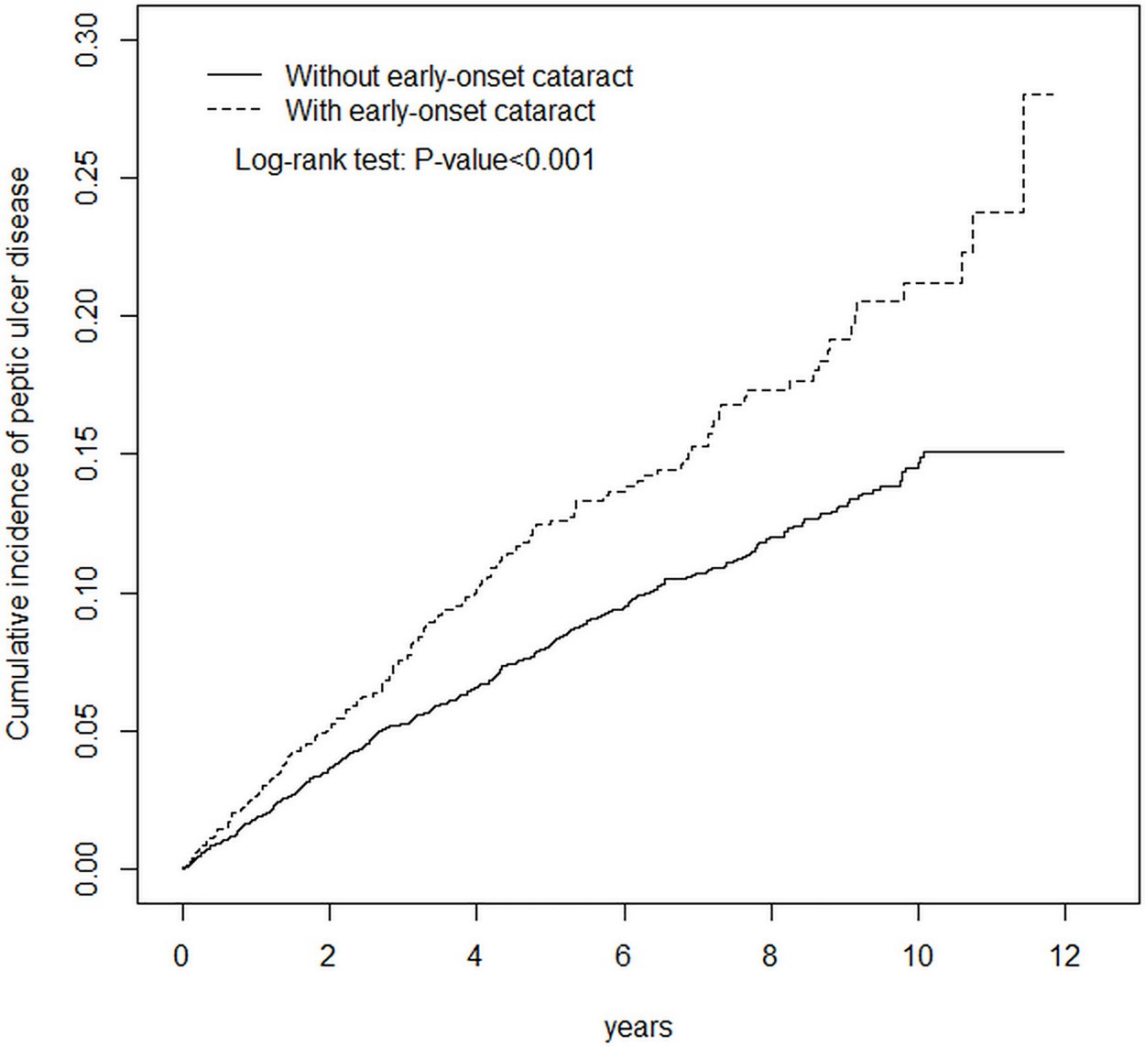
Cummulative incidence of peptic ulcer compared between patients with and without EOC. Patients with EOC had a higher cumulative incidence of peptic ulcer than those without EOC.

In Fig 1, patients with EOC had a higher cumulative incidence of peptic ulcer disease than those without EOC.

## Discussion

A significantly greater risk of subsequent peptic ulcer development among EOC patients than in the general population was found in the present retrospective cohort study. No previous study has reported the relationship between EOC and the increased risk of peptic ulcer.

There are possible mechanisms for the association between EOC and peptic ulcer, including inflammation and oxidative stress.

The association with comorbidities revealed that subjects with cataract had a higher risk of developing hypertension, coronary artery disease, and alcohol-related illness than the comparison group, as reported in many previous studies [21–24]. The underlying mechanism seems to involve oxidative stress, insufficient anti-oxidative function, glycation product formation and increased systemic inflammatory activity.

In addition to increased systemic inflammatory activity, insufficient anti-oxidative function is the cause of cataract formation. Reduced glutathione (GSH) is the most important and main antioxidant molecule in the lens. If the synthesis and recycling of GSH falls, a rise in its oxidized form (GSSH) would happen [12]. The patients with EOC have the relatively low ratio of GSH to protein-SH in the nucleus of the lens, combined with low activity of the GSH redox cycle, leading to the nucleus more vulnerable to oxidative stress [25]. Therefore, the patients with EOC may have relatively systemic poor anti-oxidation ability, so are especially vulnerable to oxidative stress and had higher risk of peptic ulcer development.

Peptic ulcer disease is a common medical condition [26, 27], and the associated complications present a substantial medical and economic burden to the healthcare system [28, 29] Two major risk factors exist for peptic ulcer disease–Helicobacter pylori infection and non-steroidal anti-inflammatory drugs (NSAIDs) use. *Helicobacter pylori* incites an inflammatory process and pro-inflammatory cytokine production in the gastroduodenal tissue and leads to chronic gastritis, which is asymptomatic in most individuals and does not progress [30–32]. Many virulence factors contribute to the pathogenesis of *H*. *pylori* infection, and cytotoxin-associated gene antigen (Cag A) plays a key role. Cag A induces an immune response via the Nox-1 pathway and IL-8/neutrophil pathway, which results in oxidative stress in the *H*. *pylori*-infected cells [33]. If this irritation lasts for a long time, while at the same time the healing capacity and anti-oxidative function are overwhelmed, the tissue will be injured and progress to peptic ulcer or even gastric cancer.

Another major risk factor is the use of NSAIDs, including low-dose aspirin. These medications are effective and widely used, but can cause peptic ulcers, which may lead to fatal complications. Our study results indicate that we should be vigilant regarding those patients with EOC and those who need long-term use of NSAIDs or low-dose aspirin treatment. Lifestyle adjustment, such as ceasing cigarette smoking and alcohol drinking, is also suggested for patients with EOC, so as to decrease the risk of developing peptic ulcer.

Our study also revealed that patients with EOC under systemic steroid treatment is associated with an increased risk of peptic ulcer and it is compatible with a recent large meta‐analysis [34].

Our strength of this study is that the source of patients was based on nationwide population-based data, which covered > 98% of the population in Taiwan and decreased the selection bias. The longitudinal study was used to monitor peptic ulcer development between the EOC and non-EOC cohorts more effectively than medical chart review or cross-sectional studies. Moreover, a complete program to verify this dataset was addressed previously, it concluded that a single-payer system can gather comprehensive information on patients and providers [35].

However, our study has a few limitations. First, detailed information about disease severity, with respect to EOC, peptic ulcer and other comorbid medical illnesses, was not available from the LHID 2000. Second, information on the established risk factors for peptic ulcer, such as cigarette smoking, alcohol consumption, or other lifestyle behaviors, was not available. Finally, the present study was an observational retrospective cohort study. Further prospective studies are warranted to clarify our findings and the underlying pathophysiological mechanisms of EOC and their associations with peptic ulcer development.

## Conclusions

Early-onset cataract patients were found to have a significantly increased risk of subsequent peptic ulcer development after adjusting for possible confounding factors. Insufficient anti-oxidative and anti-inflammatory function seems to be involved in the EOC and the peptic ulcer of susceptible people. Identification of risk factors of peptic ulcer could help reduce the incidence of peptic ulcer and the mortality arising from unanticipated complications.
